# Nociception-induced spatial and temporal plasticity of synaptic connection and function in the hippocampal formation of rats: a multi-electrode array recording

**DOI:** 10.1186/1744-8069-5-55

**Published:** 2009-09-22

**Authors:** Xiao-Yan Zhao, Ming-Gang Liu, Dong-Liang Yuan, Yan Wang, Ying He, Dan-Dan Wang, Xue-Feng Chen, Fu-Kang Zhang, Hua Li, Xiao-Sheng He, Jun Chen

**Affiliations:** 1 Institute for Biomedical Sciences of Pain, Capital Medical University, Beijing 100069, PR China; 2 Institute for Biomedical Sciences of Pain and Institute for Functional Brain Disorders, Tangdu Hospital, The Fourth Military Medical University, Xi'an 710038, PR China; 3 Department of Neurosurgery, Xijing Hospital, The Fourth Military Medical University, Xi'an 710032, PR China

## Abstract

**Background:**

Pain is known to be processed by a complex neural network (neuromatrix) in the brain. It is hypothesized that under pathological state, persistent or chronic pain can affect various higher brain functions through ascending pathways, leading to co-morbidities or mental disability of pain. However, so far the influences of pathological pain on the higher brain functions are less clear and this may hinder the advances in pain therapy. In the current study, we studied spatiotemporal plasticity of synaptic connection and function in the hippocampal formation (HF) in response to persistent nociception.

**Results:**

On the hippocampal slices of rats which had suffered from persistent nociception for 2 h by receiving subcutaneous bee venom (BV) or formalin injection into one hand paw, multisite recordings were performed by an 8 × 8 multi-electrode array probe. The waveform of the field excitatory postsynaptic potential (fEPSP), induced by perforant path electrical stimulation and pharmacologically identified as being activity-dependent and mediated by ionotropic glutamate receptors, was consistently positive-going in the dentate gyrus (DG), while that in the CA1 was negative-going in shape in naïve and saline control groups. For the spatial characteristics of synaptic plasticity, BV- or formalin-induced persistent pain significantly increased the number of detectable fEPSP in both DG and CA1 area, implicating enlargement of the synaptic connection size by the injury or acute inflammation. Moreover, the input-output function of synaptic efficacy was shown to be distinctly enhanced by the injury with the stimulus-response curve being moved leftward compared to the control. For the temporal plasticity, long-term potentiation produced by theta burst stimulation (TBS) conditioning was also remarkably enhanced by pain. Moreover, it is strikingly noted that the shape of fEPSP waveform was drastically deformed or split by a TBS conditioning under the condition of persistent nociception, while that in naïve or saline control state was not affected. All these changes in synaptic connection and function, confirmed by the 2-dimentional current source density imaging, were found to be highly correlated with peripheral persistent nociception since pre-blockade of nociceptive impulses could eliminate all of them. Finally, the initial pharmacological investigation showed that AMPA/KA glutamate receptors might play more important roles in mediation of pain-associated spatiotemporal plasticity than NMDA receptors.

**Conclusion:**

Peripheral persistent nociception produces great impact upon the higher brain structures that lead to not only temporal plasticity, but also spatial plasticity of synaptic connection and function in the HF. The spatial plasticity of synaptic activities is more complex than the temporal plasticity, comprising of enlargement of synaptic connection size at network level, deformed fEPSP at local circuit level and, increased synaptic efficacy at cellular level. In addition, the multi-synaptic model established in the present investigation may open a new avenue for future studies of pain-related brain dysfunctions at the higher level of the neuromatrix.

## Background

It has been gradually known that pain is a complex experience consisting of sensory-discriminative, affective-motivational, and cognitive-evaluative dimensions [1,2]. Furthermore, there is now a consensus of idea that noxious information is processed by a distributed and interconnected neural network, referred to as neuromatrix, in the brain [3-6]. Unlike physiological state, pathological pain, when becomes persistent or chronic, can affect various higher brain functions (such as perception, emotion, cognition, and memory) through ascending pain pathways, leading to consequences of cognitive decline and mental disability. In the past three decades, the most advanced understanding about pain is that pathogenesis or chronicity of pain is attributable to sensitization of primary sensory neurons and synaptic plasticity in dorsal horn of the spinal cord [7-9]. To date, the mechanisms by which inflammatory or neuropathic pain is processed at the lower level of the pain pathway have been well characterized [7-11]. However, in clinic, chronic pain often results in not only sensory dysfunction (spontaneous pain, hyperalgesia and allodynia, etc.) but also emotional and cognitive disorders such as anxiety, amnesia and depression [12-14]. Unfortunately, so far, the influences of pathological pain on the higher brain functions are not clear and this may hinder the advances in clinical pain therapy. Therefore, unraveling how pain affects the emotion- or cognition-controlling regions at a higher level of the "pain matrix" would definitely improve our understanding of the process of pain chronicity and provide novel strategies for treating negative emotional symptoms of chronic pain in the clinical setting [4,6].

There is substantial evidence indicating that the hippocampal formation (HF), an integral component of the limbic system [15,16], is involved in pain processing besides its well documented roles in learning and memory formation [17-19]. Melzack and Casey (1968) proposed that the limbic forebrain structures, including the HF, play important roles in the 'aversive drive and affect that comprise the motivational dimension of pain' [20]. Anatomically, the HF is positioned as a key interconnecting structure in Papez's circuit of the limbic system, mediating a variety of biological functions, including learning and memory, anxiety, emotion and sensorimotor integration [15,21,22]. More recent evidence using the atlas registration-based event-related (ARBER) analysis technique and whole-brain functional magnetic resonance (fMRI) imaging or 18F-fluorodeoxyglucose positron emission tomography (PET) clearly shows that dorsal, but not ventral, part of the rat HF was activated by subcutaneous formalin injection [23,24]. Previous studies using electrophysiological [25-31] and neurochemical/biochemical [32-37] assays have demonstrated that the neuronal activities (pyramidal or interneuronal) and protein expression/activation within the HF could be altered by pain and stress. Moreover, intra-hippocampal microinjection of lidocaine [38], or antagonists acting at N-methyl-D-aspartic acid (NMDA) receptor [39,40], 5-HT_2A/2C _receptor [41] and platelet-activating factor receptor [42] could result in an analgesic effect in the formalin test. Clinical observations show that electrical stimulation of the HF evokes painful sensations in humans [43,44] and hippocampal lesion can partially alleviate chronic pain [45,46]. Taken together, the above previous reports provide convergent evidence for the critical involvement of HF in pain processing and support the possibility that there might be some kinds of synaptic plasticity occurred in the HF characterized by functional changes in synaptic transmission and modulation as well as structural changes in synaptic connection under the condition of peripheral persistent nociception.

With regard to the impact of pain upon the brain, it has been revealed that chronic pain states can change the structure and morphology of the brain, namely central structural plasticity, which probably results in long-term dysfunction of synaptic transmission and modulation at different levels of the central nervous system (CNS) [47]. In addition, long-term potentiation (LTP), a form of functional neuroplasticity, was also found to be associated with pain processing in recent years, except for its wide use as a unique synaptic model for learning and memory [48-50]. Actually, there have been a number of previous studies investigating LTP phenomenon in multiple pain-related CNS regions, including the spinal cord dorsal horn [51-53], primary somatosensory cortex (S1 area) [54,55], amygdala [55,56], anterior cingulate cortex [55,57-61] and so on. As regards the HF, an enhanced LTP by pain was also reported in one previous study [62]. In that study, it was found that LTP in the CA1 area could be facilitated by tail tip amputation-induced injury in mice and the increased synaptic efficacy was accompanied by a strong up-regulation of the immediate early gene product Egr1 [62]. Because CA1 receives inputs from Schaffer collaterals of CA3 pyramidal cells which are innervated by entorhinal-dentate gyrus (DG) output [15,16,22], it is of particular importance to see whether there are parallel changes in synaptic connection and function in the DG area in response to persistent nociception. Furthermore, based upon the studies from Khanna's group, the neuronal activities in CA1 differ from each other in response to formalin-induced nociception, namely at the time when a discrete population of putative pyramidal cells are selectively activated, a large number of CA1 cells are suppressed in a widespread and prolonged manner, implicating a 'signal-to-noise' processing of pain in the CA1 area [27-31]. However, the interrelationship between different populations of CA1 neurons or between DG and CA1 regions are still not clear and requires to be further studied by multisite recording approaches.

The planar multi-electrode array (pMEA) is a unique and well-established tool for investigating, at a macroscopic level, the electrophysiological properties of living brain slices containing intact networks of neurons, providing a bridge between single cell testing and behavioral studies [63]. Compared to traditional electrophysiology, the pMEA technique allows one to detect the activity of neuronal networks in both space and time [63-65], to record multiple sites simultaneously [66], and to make stimulating the recorded cells possible [67]. To visualize the spatial and temporal information of pMEA recording, two-dimensional current source density (2D-CSD) imaging can also be used [68,69]. Therefore, in the present study, using pMEA (i.e., Panasonic's MED64 system, see [68-70]) recordings combined with 2D-CSD imaging on acute hippocampal slices, we examined potential effects of peripheral persistent nociception on spatial and temporal plasticity of synaptic connection and function in the HF. The animal pain models we used are the bee venom (BV) test and the formalin test, both of them being well-developed animal models of persistent, inflammatory pain [71-76]. The results showed robust changes in both spatial and temporal plasticity of synaptic connection and function following peripheral persistent nociception.

## Results

### Two types of field potentials were recorded in the HF

**Figure 1:**
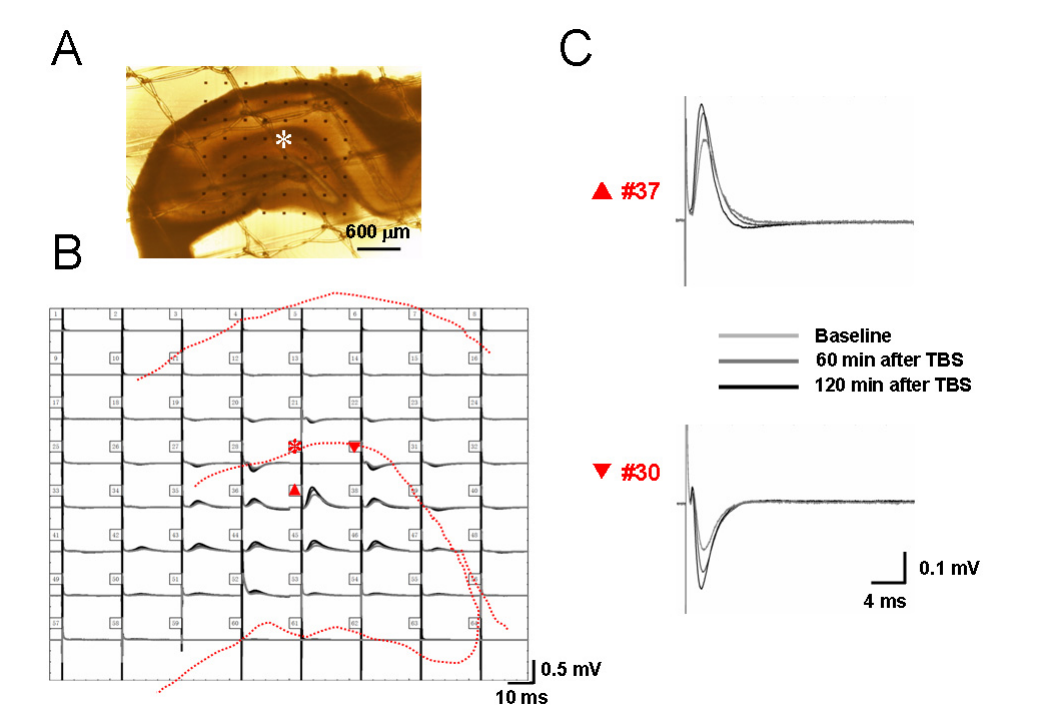
**Showing a typical example of the MED64 probe recordings on the hippocampal slice of a rat receiving saline injection into a hindpaw**. A, a photograph showing one hippocampal formation (HF) positioned on a Med64 probe with 8 × 8 arrays (interelectrode distance: 300 μm). The asterisk indicates an electrode selected for electrical stimulation of perforant path (PP) fibers. The dorsal part of the HF corresponds to the top of the image, and the lateral side of the HF is shown at left of the image. B, real traces of 63 recording electrodes across the dentate gyrus (DG) and the CA1 area in response to the PP test stimulation before, 60 min and 120 min after theta burst conditioning stimulation (TBS). Dashed lines indicate the anatomical contour of the HF. C, example field potentials of the electrodes #37 and #30 (indicated by upward and reverse arrows in B) were shown to be positive-going (upper) in the DG and negative-going (lower) in the CA1. The amplitude of the field potentials in both areas was potentiated for a long-term period after TBS conditioning of the PP fibers (asterisk in B). Scale bar in A: 600 μm; Vertical scale in both B and C indicates amplitude of the potentials, while horizontal scale indicates time sweep.

**Figure 2:**
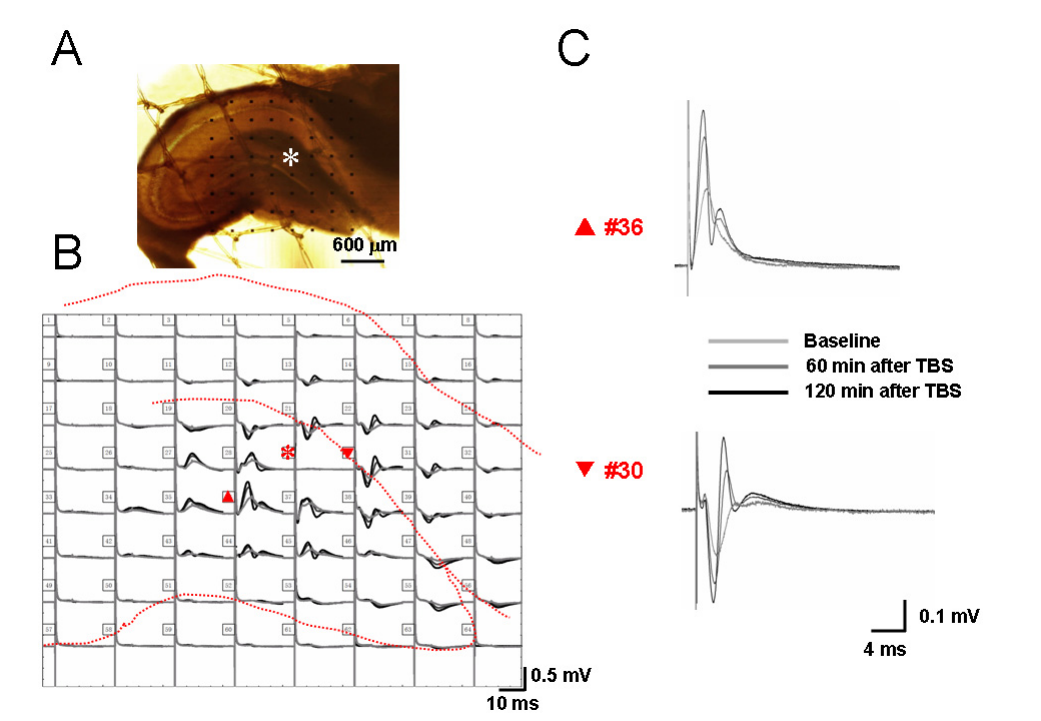
**Showing a typical example of the MED64 probe recordings on the hippocampal slice of a rat receiving bee venom injection into a hindpaw**. A, a photograph showing one hippocampal formation (HF) positioned on a Med64 probe with 8 × 8 arrays (interelectrode distance: 300 μm). The asterisk indicates an electrode selected for electrical stimulation of perforant path (PP) fibers. The dorsal part of the HF corresponds to the top of the image, and the lateral side of the HF is shown at left of the image. B, real traces of 63 recording electrodes across the dentate gyrus (DG) and the CA1 area in response to the PP test stimulation before, 60 min and 120 min after theta burst conditioning stimulation (TBS). Dashed lines indicate the anatomical contour of the HF. C, example field potentials of the electrodes #36 and #30 (indicated by upward and reverse arrows in B) were shown to be positive-going (upper) in the DG and negative-going (lower) in the CA1. The shape of the field potentials was deformed or split after TBS conditioning. The amplitude of the field potentials in both areas was appreciably potentiated for a long-term period after TBS conditioning of the PP fibers (asterisk in B). Scale bar in A: 600 μm; Vertical scale in both B and C indicates amplitude of the potentials, while horizontal scale indicates time sweep.

Under the current experimental conditions by using the 8 × 8 multi-electrode probe, we found that electrical stimulation of the perforant path (PP), one major input from the entorhinal area, typically evoked two kinds of characteristic field potentials. In the DG, a positive-going waveform was consistently observed, while in the area corresponding to stratum lacunosum-moleculare of the CA1 region, a negative-going response was uniformly detected (for examples, see Fig. 1 and 2). Most of these electrical responses exhibited single phase in the appearance. On average, the latency for the peak of positive- and negative-going field potentials was 2-4 ms. This phenomenon was reliably observed in most of the slices examined and reflected electrophysiological properties of ensembles of neurons acting together as a network. Those slices that did not meet this criterion were excluded from the final analysis.

**Figure 3:**
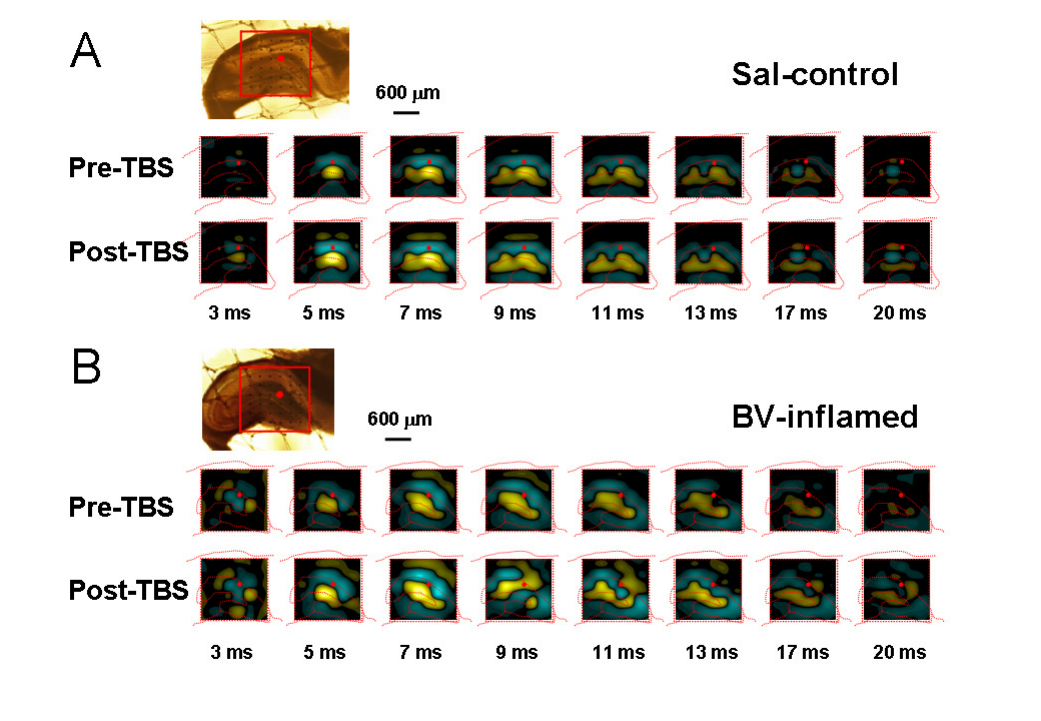
**Showing two-dimensional current source density (2D-CSD) imaging of 8 × 8 array (inset, interelectrode spacing: 300 μm, scale bar: 600 μm) recordings in the hippocampal formation**. A, an example of 2D-CSD imaging of network response across the dentate gyrus (DG) and CA1 area to theta burst stimulation (TBS) of the perforant path (PP) on the hippocampal slice of a rat receiving saline injection (Sal-control). B, an example of 2D-CSD imaging of network responses across the DG and CA1 area to the PP TBS conditioning on the hippocampal slice of a rat receiving bee venom injection (BV-inflamed). Recordings were taken from 63 sites in the hippocampal slice in response to PP electrical stimulation (red dots). Each imaging represents the instantaneous 2D-CSD plots computed at selected time points, indicated at the bottom of each column. Current sinks are depicted in blue and current sources are shown in yellow. The positions of the pyramidal and granule cell bodies are marked by red, dashed lines. Pre-TBS, baseline; Post-TBS, 120 min after TBS conditioning. Note the conversion from single current source-sink dipole to binomial events after TBS in the BV-inflamed group.

To visualize the multisite-recorded neural responses more vividly, we generated movies of neuronal activities within the hippocampal circuit on a millisecond time scale resolution by performing 2D-CSD imaging on acute hippocampal slices. The 2D-CSD analysis is an analyzing method often used to estimate the location and distribution of synaptic currents underlying recorded field potentials [77]. It can, at first, alleviate the problems of volume conduction and low spatial resolution associated with multi-electrode recording [66]. Second, all signals generated on the MED64 probe can be transformed into one coherent image for each time point sampled, revealing spatiotemporal aspects of current movements in brain slices [68]. Fig. 3 illustrates the results of 2D-CSD analysis for selected sweep time points (indicated at the bottom of each column) following stimulation of PP fibers. The outlines of the HF area were visible (red, dashed lines) in each frame. The computed sink-source series obtained from saline-treated slices corresponded closely to the time course and magnitude of the original waveforms (see Fig. 1 and Fig. 3A, upper row). After an initial response due to the fiber volley (3 ms), a current source (yellow) emerged in the DG at 5 ms. The source intensified rapidly (7 ms), expanded over roughly 3 ms (8-10 ms), and then faded (11 ms), disappearing at about 20 ms. The current source detected in the DG region was consistently accompanied by a field of reversed polarity (current sink, blue) in the CA1 area, which grew and dissipated with almost the same time course as the DG events. To summarize, the prominent field potential evoked in the saline control group could be characterized as a current source-sink dipole occurring mainly from 5 to 11 ms. Simultaneous CSD images computed after 20 ms were not discussed here due to a large degree of temporal variability in estimated currents developed across slices.

### Pharmacological identification of two kinds of field potentials

**Figure 4:**
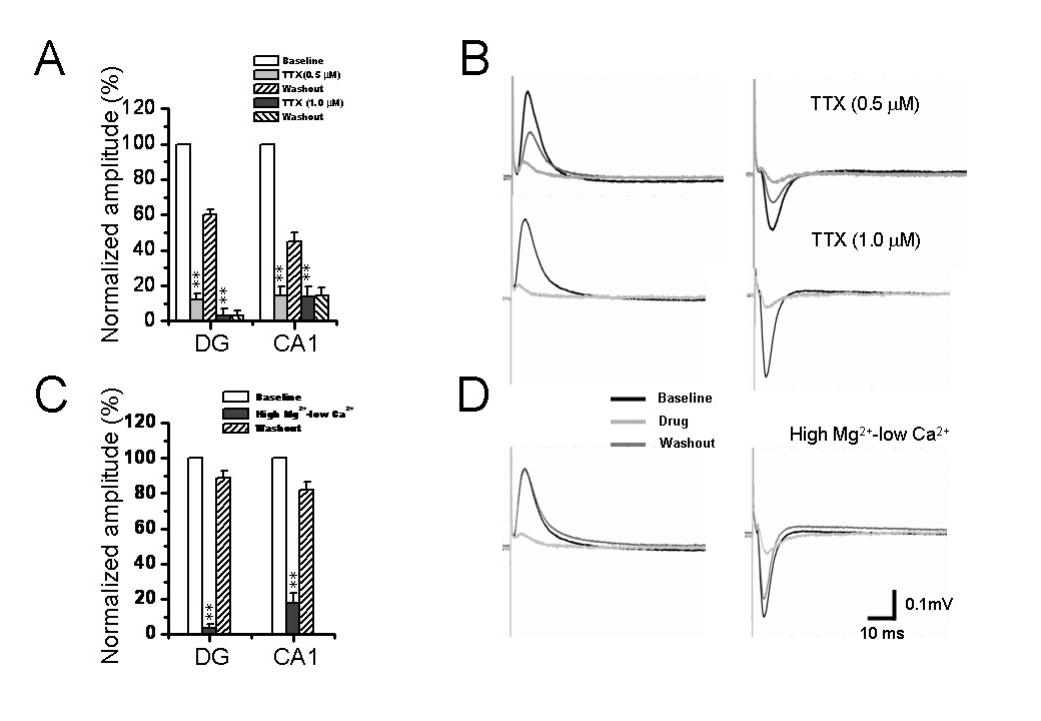
**Pharmacological identification of the perforant path-induced network field potentials on the hippocampal slices**. A and C, showing pooled data from 7 similar experiments with strong inhibition of field potentials by either bath application of TTX (0.5 μM and 1 μM) or perfusion with high magnesium-low calcium solution (CaCl_2_, 0.25 mM, MgSO_4_, 4.0 mM). All values were normalized as a percentage of baseline. B and D, showing representative raw traces of positive- (left) and negative-going (right) field potentials recorded in the dentate gyrus and CA1 area before treatment, 10 min after TTX infusion (B) or ionic substitution (D), and the washout end. Vertical scale indicates amplitude of the potentials, while horizontal scale indicates time sweep for both B and D. **P < 0.01 vs. baseline. Error bars: ± S.E.M.

To verify that the field potentials recorded by our MED64 electrodes were mediated by synaptic connection and transmission, we performed a series of experiments using pharmacological compounds and ionic substitutions. Perfusion of the slices with the selective fast sodium channel antagonist tetrodotoxin (TTX, 1 μM) resulted in a dramatic and irreversible decrease in the field potential amplitude (96.90 ± 3.71% for DG and 86.00 ± 5.65% for CA1, n = 7, *P *< 0.01, Fig. 4A), an effect that could be attributed to the inhibition of spike propagation along the PP axons. Notably, if the concentration of TTX was lowered to 0.5 μM, the resultant inhibition of field potentials became partially reversible (Fig. 4A), thus excluding the possibility that the observed decrease in the potential amplitude was due to a destabilized state or decreased viability of the slice with prolongation of the recording period. Representative examples of TTX-evoked inhibition at the two doses were shown in Fig. 4B for both DG (left panel) and CA1 (right panel) potentials. When the slice was perfused with a high magnesium-low calcium solution, a classical protocol to decrease neurotransmitter release [78], the amplitude of field potentials was also strongly suppressed by 96.40 ± 2.36% (n = 7, *P *< 0.01) and 82.33 ± 5.96% (n = 7, *P *< 0.01) for positive- and negative-going field potentials, respectively (Fig. 4C and 4D). Combining the results from TTX blockade and ionic substitution, we conclude at this point that the multisite neural responses recorded under our conditions are activity-dependent, relying on both action potential propagation and Ca^2+^-associated transmitter release from nerve terminals following electrical stimulation of PP fibers.

**Figure 5:**
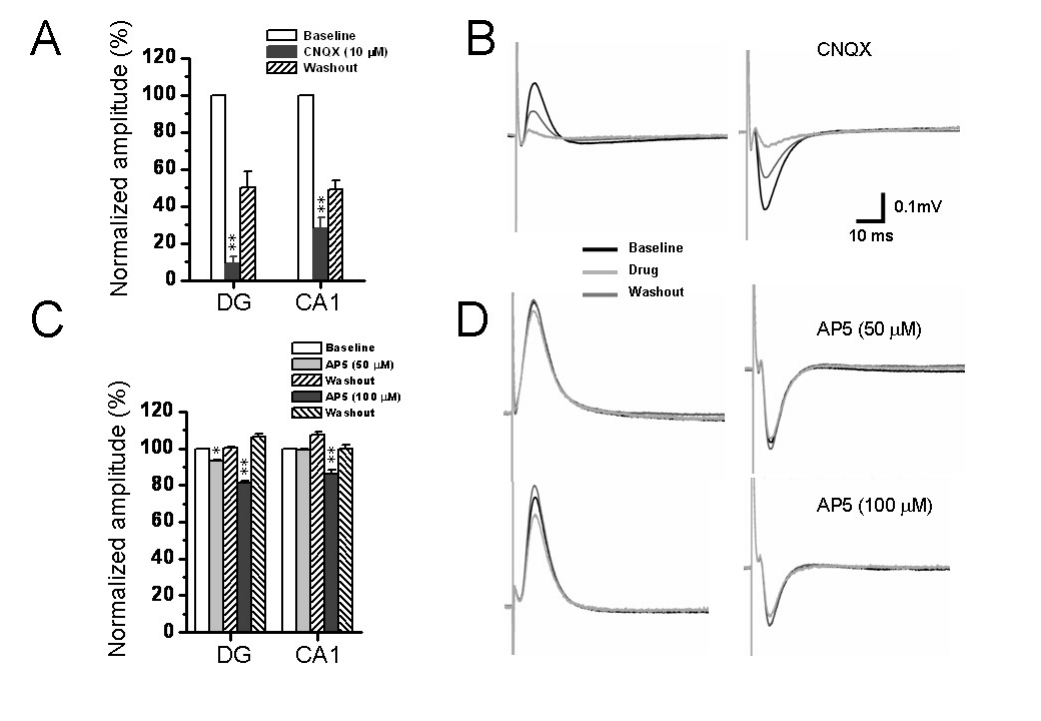
**Pharmacological identification of the perforant path-induced network field potentials on the hippocampal slices**. A and C, showing pooled data from 7 similar experiments with strong inhibition of field potentials by bath application of CNQX (10 μM) and less suppression by perfusion with AP5 (50 μM and 100 μM). All values were normalized as a percentage of baseline. B and D, showing representative raw traces of positive- (left) and negative-going (right) field potentials recorded in the dentate gyrus and CA1 area before treatment, 10 min after CNQX (B) or AP5 (D) infusion, and the washout end. Vertical scale indicates amplitude of the potentials, while horizontal scale indicates time sweep for both B and D. *P < 0.05, **P < 0.01 vs. baseline. Error bars: ± S.E.M.

Because it has been well known that the PP fibers are glutamatergic, we next clarified the postsynaptic receptor types mediating the two kinds of field potentials. In this section of experiments, effects of D, L-2-amino-5-phosphonopentanoic acid (AP5) or 6-cyano-7-nitroquinoxaline-2, 3-dione (CNQX), antagonists acting on NMDA or AMPA/KA (alpha-amino-3-hydroxy-5-methyl-4-isoxazolepropionate/kainate) receptors, were tested. Pooled results from seven individual experiments were shown in Fig. 5A and 5C for CNQX and AP5, respectively. Example experiments were illustrated in Fig. 5B and 5D. The amplitude of DG field potentials was slightly, but significantly, attenuated by 50 μM AP5 (6.93 ± 0.85%, n = 7, *P *< 0.05) and completely abolished by 10 μM CNQX (91.01 ± 3.73%, n = 7, *P *< 0.01). However, for the CA1 field potentials, 50 μM AP5 failed to cause any significant inhibitory effect (0.83 ± 0.54%, n = 7, *P *> 0.05), but 10 μM CNQX resulted in 71.93 ± 5.68% suppression (n = 7, *P *< 0.01). Furthermore, when the concentration of AP5 was raised to 100 μM, the consequent inhibition was increased, but it was still much smaller than that evoked by 10 μM CNQX (Fig. 5A and 5C). These results indicate that the field potentials recorded from both DG and CA1 area belong to one kind of field excitatory postsynaptic potential (fEPSP) principally mediated by ionotropic glutamate receptors, with AMPA/KA receptors comprising the main component and NMDA receptors partially involved. Moreover, the pharmacological results suggest existence of a difference in distribution of NMDA receptors between the DG granular cell dendrites and the CA1 pyramidal cell apical dendrites (see Discussion).

### Persistent pain produced spatial plasticity of synaptic connection and transmission in the HF

**Figure 6:**
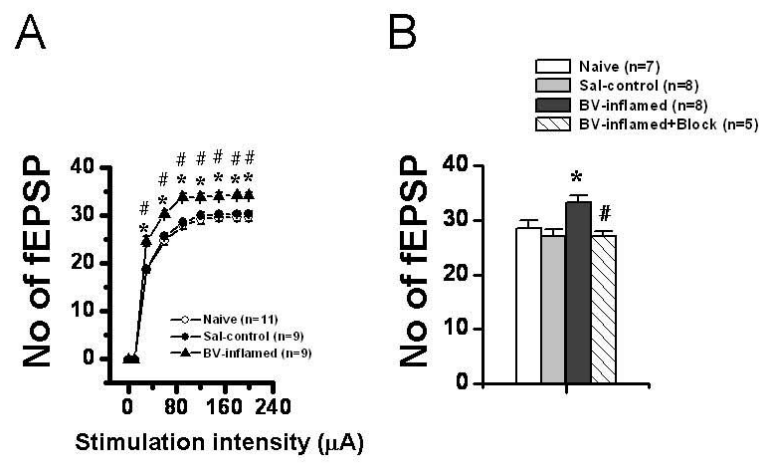
**Showing quantitative analysis of spatial plasticity of synaptic connection size in the hippocampal formation following bee venom (BV)-induced persistent nociception**. A, stimulus intensity-network response functional curves showing the mean averaged number of field excitatory postsynaptic potential (fEPSP) that was reliably evoked (>20% baseline) across the 8 × 8 arrays with the increasing stimulus intensity in naïve, saline (Sal-control), and BV-inflamed group of slices. Note the significant increase in the number of fEPSP following BV-induced persistent pain with each suprathreshold stimulus intensity applied (30-199 μA). The number of slices for each group is shown in parentheses. *P < 0.05 vs. naïve control; #*P *< 0.05 vs. saline control. Error bars: ± S.E.M. B, effects of peripheral nerve impulses blockade on the BV-induced spatial plasticity of synaptic connection. Peripheral bupivacaine pre-treatment fully abolished BV-elicited increase of the number of fEPSP. *P < 0.05 vs. saline control or naïve; #*P *< 0.05 vs. BV-inflamed. Error bars: ± S.E.M.

**Figure 7:**
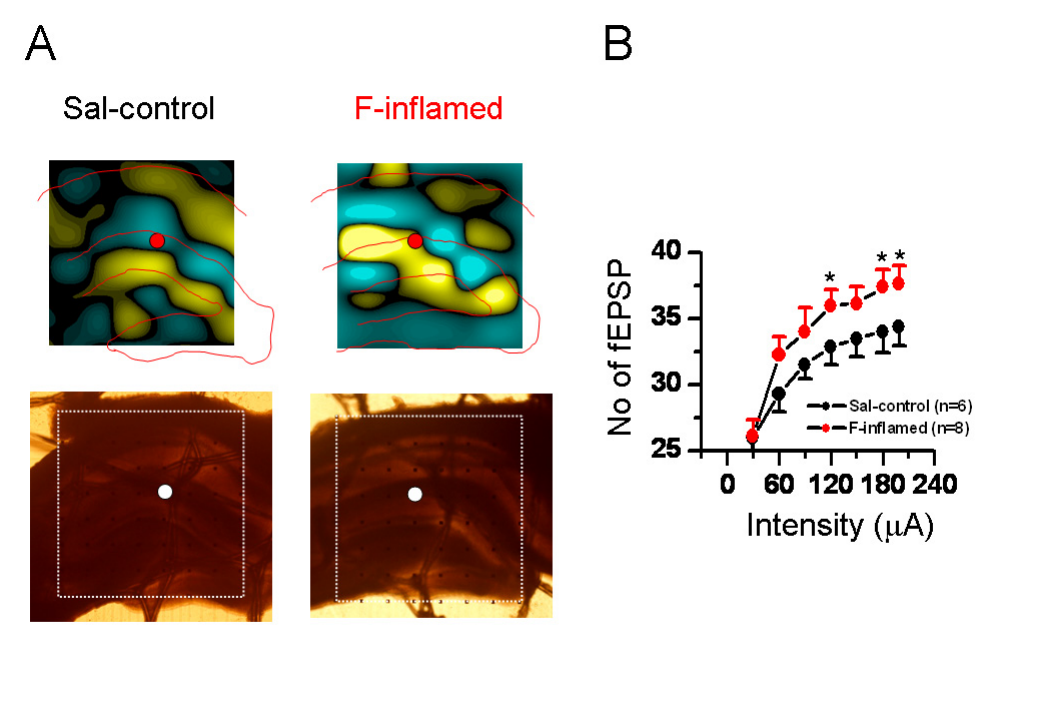
**Showing two-dimensional current source density (2D-CSD) imaging and quantitative analysis of spatial plasticity of synaptic connection in the hippocampal formation (HF) following formalin (F)-induced persistent pain**. A, the 2D-CSD images of current sources and sinks around the HF in response to electrical stimulation of the perforant pathway at an intensity of 60 μA (about half of the maximum amplitude). The lower two pictures are the original hippocampal slices from which the CSD plots were derived. For other legends, see Fig. 3. B, stimulus intensity-network response functional curves showing the mean averaged number of field excitatory postsynaptic potential (fEPSP) that was reliably evoked (>20% baseline) across the 8 × 8 arrays with the increasing stimulus intensity in saline (Sal-control), and F-inflamed group of slices. Note the significant increase in the number of fEPSP following F-induced persistent pain at the stimulation intensity of 120 μA, 180 μA and 199 μA. The number of slices for each group is shown in parentheses. *P < 0.05 vs. saline control. Error bars: ± S.E.M.

The spatial properties of synaptic connection in the HF were studied by counting the mean number of effective fEPSP (> 20% of baseline), including both positive-going and negative-going waveforms, which could be reliably recorded across the entire 64-recording screen. We constructed an input-output (I-O) curve with the number of fEPSPs plotted as a function of serial stimulus intensities within the range of 30-199 μA (Fig. 6A, Fig. 7B). The averaged threshold to evoke a detectable fEPSP was around 10-12 μA across all groups.

When examining the effects of BV-induced persistent pain on this kind of spatial plasticity, the mean total number of effective fEPSP was robustly increased in hippocampal slices prepared from rats receiving intraplantar injection of BV when compared to those from naïve or saline-treated animals at each stimulus applied. No appreciable difference was detected between saline control and naïve group (Fig. 6A). Within each group, the number rose gradually with increasing stimulus intensity and reached a stable level at around 90-120 μA. Locally pre-administration of an anesthetic agent (0.25% bupivacaine, 10 min prior to BV injection) resulted in an almost complete blockade of BV-induced increase in the number of fEPSP at a certain stimulation intensity (40-60% of the maximal response, 33.21 ± 1.25 vs. 27.00 ± 0.95 for BV-inflamed vs. BV-inflamed + block, n = 8 and 5, *P *< 0.05, Fig. 6B).

Almost similar results were obtained in the formalin test. As shown in Fig. 7B, compared with the saline control group, subcutaneous injection of 5% formalin solution elicited a larger synaptic connection size over the HF, reflected as the significantly increased number of effective fEPSP at the stimulation intensity of 120 μA, 180 μA and 199 μA.

**Figure 8:**
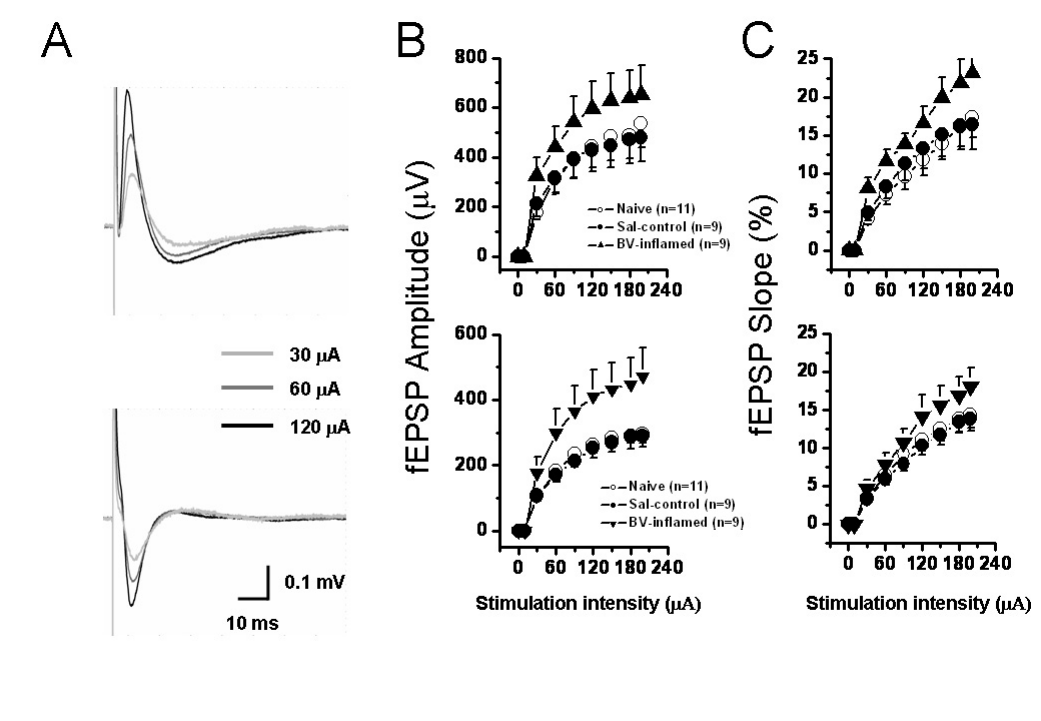
**Showing stimulus intensity-network response functional curves in the hippocampal formation of naïve, saline (Sal-control) and bee venom (BV)-inflamed rats**. A, an example showing that individual field excitatory postsynaptic potential in either the dentate gyrus (DG) (upper) or the CA1 (lower) area was increased in amplitude (B) or slope (C) in an intensity-dependent manner. The input-output functional curves of the DG (upper) and CA1 (lower) network response were leftward shifted in the BV-inflamed rats to those of naïve and Sal-control rats. Vertical scale in A indicates amplitude of the potentials, while horizontal scale indicates time sweep. The number of slices for each group is shown in parentheses. Error bars: ± S.E.M.

**Figure 9:**
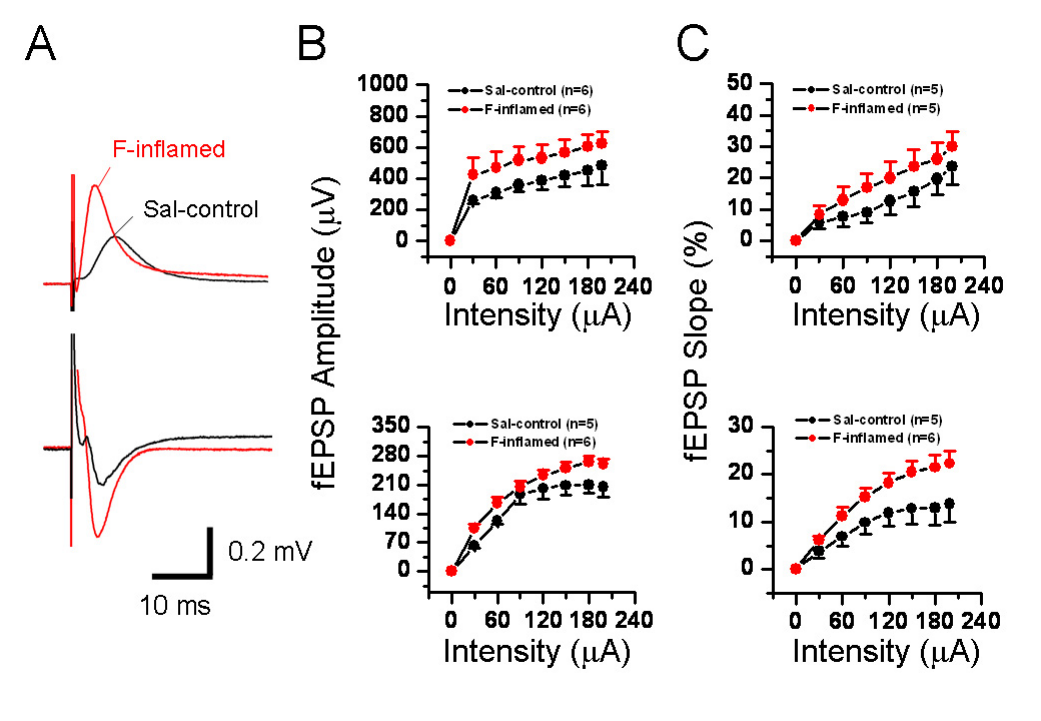
**Showing stimulus intensity-network response functional curves in the hippocampal formation of saline (Sal-control) and formalin (F)-inflamed rats**. A, an example showing the individual field excitatory postsynaptic potentials (fEPSPs) in either the dentate gyrus (DG) (upper) or the CA1 (lower) area of Sal-control and F-inflamed slices. Input/output relationships of stimulus intensity versus amplitude (B) or (C) slope of DG- (upper) and CA1 (lower) fEPSP were constructed. Note the formalin-induced significant leftward shift of input/output curves for fEPSP amplitude and slope. Vertical scale in A indicates amplitude of the potentials, while horizontal scale indicates time sweep. The number of slices for each group is shown in parentheses. Error bars: ± S.E.M.

The above-described results were mainly focusing on spatial plasticity of synaptic connection within the whole recording screen. Subsequently, we asked whether the same series of graded stimulus intensities may produce spatial summation of electrical activity of one site among the 64 electrodes. Similarly, we established several I-O functional curves of multi-synaptic transmission in terms of either amplitude or slope of the evoked potentials (Fig. 8 and 9). Sample traces of fEPSP recorded at three different intensities (30 μA, 60 μA, and 120 μA) were illustrated in Fig. 8A for DG (upper panel) and CA1 (lower panel) fEPSP. As delineated in Fig. 8B, the I-O relationships of the amplitude of fEPSP (both DG and CA1) exhibited an apparent left-ward shift in the BV-inflamed group, although the curves of the saline control group were indistinguishable from those of the naïve slices. This trend was less remarkable, but still present, in the case of the slope of fEPSP (Fig. 8C). Persistent pain induced by subcutaneous injection of formalin also caused the leftward shift of these I-O curves, but the shift was much less clear than that of BV-inflamed group (Fig. 9). The 2D-CSD imaging analysis further confirmed this phenomenon, showing an increase of the current signal (both sources and sinks) around the HF in the formalin-inflamed slices in comparison with the saline control (Fig. 7A). Altogether, these results implicate an enhanced synaptic responsiveness to unconditioned test electrical stimuli following BV- or formalin-induced persistent nociception. It seems necessary to claim that the I-O curves did not move left-ward in a parallel manner, indicating no alteration in the threshold for fEPSP detection.

### Persistent pain enhanced temporal plasticity of synaptic responses in the HF

**Figure 10:**
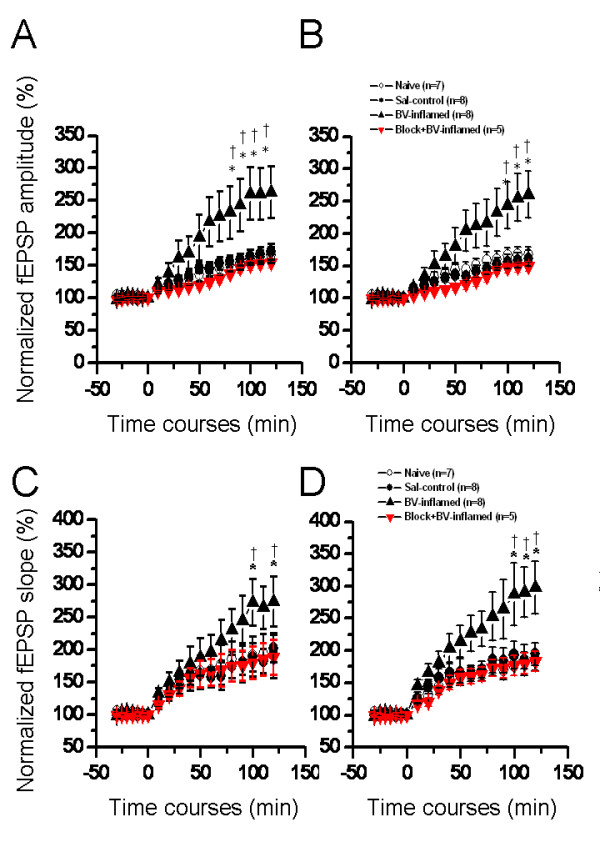
**Showing a comparison of long-term potentiation (LTP) of field excitatory postsynaptic potential (fEPSP) in the hippocampal formation induced by perforant path theta burst stimulation (TBS) conditioning between groups of rats in naïve, saline (Sal-control), bee venom (BV)-inflamed and peripheral impulse blockade state**. The amplitude (A, B) and slope (C, D) of both dentate gyrus (A, C) and CA1 (B, D) fEPSP were normalized as percentage of the pre-TBS baseline and plotted as a function of time. Enhancement of network LTP by BV-induced persistent nociception could be reversed by local pre-blockade of nerve impulses from injury site. The number of slices used to plot the graph is indicated in parentheses. *P < 0.05 vs. naïve control; †*P *< 0.05 vs. saline control. Error bars: ± S.E.M.

LTP reflects the power of synaptic function and constitutes the most widely used paradigm for study of synaptic plasticity that underlies information processing and storage in neuronal circuits [48-50]. Therefore, we subsequently examined whether characteristics of LTP evoked in the PP pathway with theta burst stimulation (TBS) could be altered following peripheral persistent nociception. Localization of stimulating (asterisk) and recording electrodes (the other 63 electrodes) beneath the hippocampal slice from one saline-treated animal was shown in Fig. 1A. A brief overview of overall synaptic responses was displayed in Fig. 1B, illustrating superposed fEPSP recorded before (baseline), 60 min after, and 120 min after TBS conditioning. It was apparent that TBS caused a significant increase in the amplitude of both positive- and negative-going fEPSP in saline control group of slices (Fig. 1B, C). Quantification of LTP induction was shown in Fig. 10. Application of TBS to the PP fibers on hippocampal slices of saline control rats led to a long-lasting (more than 2 h) increase in synaptic strength, with the normalized fEPSP amplitude at post-TBS 120 min being 171.63 ± 12.63% (n = 8) and 161.55 ± 12.46% (n = 8) for DG (Fig. 10A) and CA1 (Fig. 10B) fEPSP, respectively. Results for the induction of LTP in the naïve slices were similar to those of the saline control group (166.85 ± 12.24% and 167.43 ± 11.72% of baseline at post-TBS 120 min for DG and CA1, respectively; n = 7) and statistical analysis revealed no significant difference between the two control groups (Fig. 10A and 10B). However, on hippocampal slices of rats suffering from persistent nociception caused by subcutaneous BV injection, the magnitude of LTP was dramatically enhanced with the time period between 90-120 min (for DG fEPSP, Fig. 10A) or 100-120 min (for CA1 fEPSP, Fig. 10B) being statistically significant compared with the control. The normalized amplitude at post-TBS 120 min was 263.23 ± 39.53% (n = 8) for the DG and 260.79 ± 36.17% (n = 8) for the CA1, representing more than 1.5 fold larger than the control. Peripheral pre-administration of bupivacaine (0.25%, 10 min prior to BV injection) completely inhibited BV-induced enhancement of LTP magnitude but not profoundly impaired the LTP induction (Fig. 10A, B). Importantly, all these findings were repeated when analyzing the slope of fEPSP during LTP induction (Fig. 10C and 10D).

**Figure 11:**
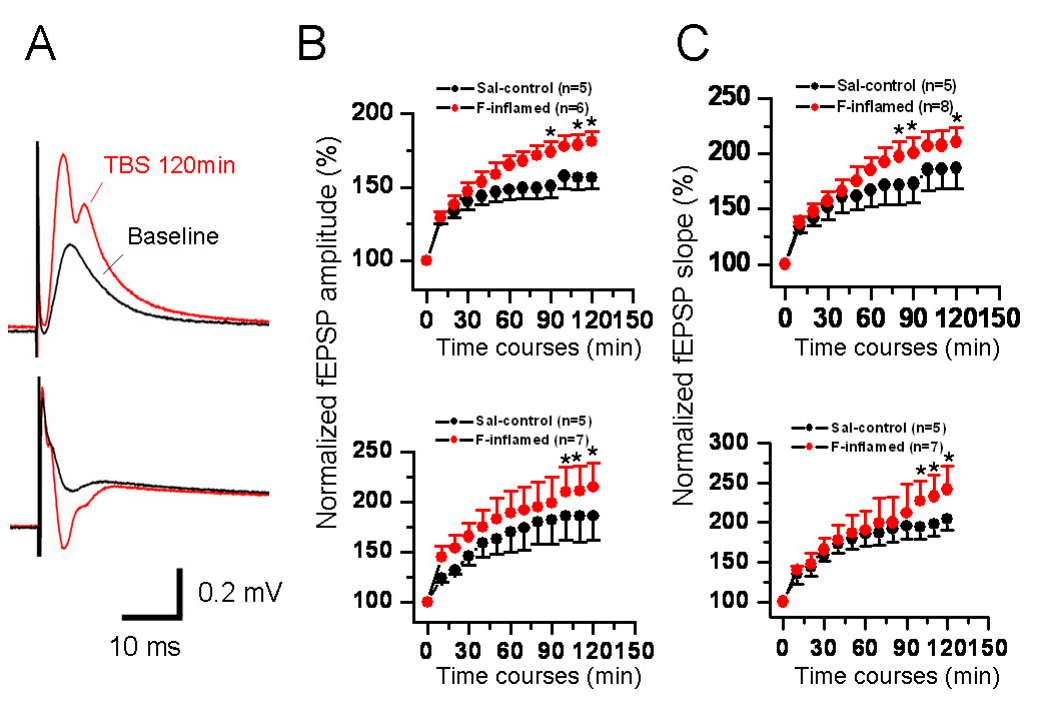
**Showing a comparison of long-term potentiation (LTP) of field excitatory postsynaptic potential (fEPSP) in the hippocampal formation induced by perforant path theta burst stimulation (TBS) conditioning between groups of rats in saline (Sal-control) and formalin (F)-inflamed state**. A, a typical example of F-induced alteration in the shape of fEPSP at 120 min after TBS. The amplitude (B) and slope (C) of both dentate gyrus (upper) and CA1 (lower) fEPSP were normalized as percentage of the pre-TBS baseline and plotted as a function of time. F-evoked persistent pain could also produce enhancement of LTP in hippocampal slices. The number of slices used to plot the graph is indicated in parentheses. *P < 0.05 vs. saline control. Error bars: ± S.E.M.

When it comes to formalin-initiated temporal plasticity, Fig. 11 also demonstrated the enhancement of LTP magnitude, in terms of either amplitude or slope of fEPSP, by formalin-evoked pain when compared with the saline control. However, it appears that formalin-induced increase in LTP magnitude was not so large as that caused by BV-induced persistent pain (compare Fig. 10 and Fig. 11).

Induction probability of LTP is also a parameter describing functional stability of facilitated synaptic efficacy that may reflect the network state of the CNS structures. Following recording of a stable baseline, LTP was successfully elicited in 16 out of 23 naïve slices, with an induction rate of 69.6%. The success rate of LTP induction was about 72.4% in the saline-treated group (21/29), which was not significantly different from the naïve control. Nevertheless, the rate was increased to 85.7% (24/28) and 83.3% (10/12) in the BV- and formalin-treated group of slices.

The waveform of the fEPSP is determined by field current direction and distribution across the synaptic interconnections due to depolarization and repolarization of the membrane. The 2D-CSD imaging is based mostly upon the spatiotemporal distribution of current sources (positive-going waveforms) and sinks (negative-going waveforms). Thus, the changes in waveforms of fEPSP will lead to changes in current sources and sinks, resulting in corresponding changes in 2D-CSD imaging. In the current study, it was surprisingly noted that, in some cases (about 50% of all the slices exhibiting LTP from BV- or formalin-inflamed rat), the shape of both positive-going and negative-going fEPSP was deformed or tortured at around 30-60 min after TBS conditioning on the hippocampal slices of rats which had suffered from persistent nociception, namely from the original single phase to double or multiple phases (Fig. 2C and Fig. 11A). These alterations in the appearance or structure of fEPSP were also reflected in the 2D-CSD plots (Fig. 3). After application of TBS onto the PP pathway, instantaneous 2D-CSD plots computed across all 64 electrodes revealed a clear rise in the intensity of current signals detected from slices of the saline-treated group (Fig. 3A, lower row). In naïve slices, 2D-CSD images constructed before and after LTP induction were similar to those from the saline group (data not shown). Unlike naïve or saline-treated slices, plastic alterations in 2D-CSD images following BV-induced persistent pain seemed rather complex. While the pre-TBS profile was essentially the same as that described above (Fig. 3B, upper row), post-TBS pictures (Fig. 3B, lower row) revealed a dramatic and interesting change in the spatial distribution of current sinks and sources, from single dipole to binomial events. Specifically, the initial fiber volley was followed by the growth of a current source in the DG region, accompanied by a current sink in the CA1 area (see image at 5 ms). The sinks and sources then increased in density, extended in scope, and reached a peak at 7 ms. However, by 9 ms, currents on both the DG and CA1 became a mixture of sources and sinks. This state persisted for the next 10 ms before vanishing around 20 ms. Moreover, tetanization of the PP pathway produced a more robust increase in the intensity of CSD signals, with the disparity between post-TBS and pre-TBS being much larger in the BV-treated group than the others (Fig. 3).

### Roles of NMDA and AMPA/KA receptors in mediation of persistent nociception-induced spatial and temporal plasticity in the HF

**Figure 12:**
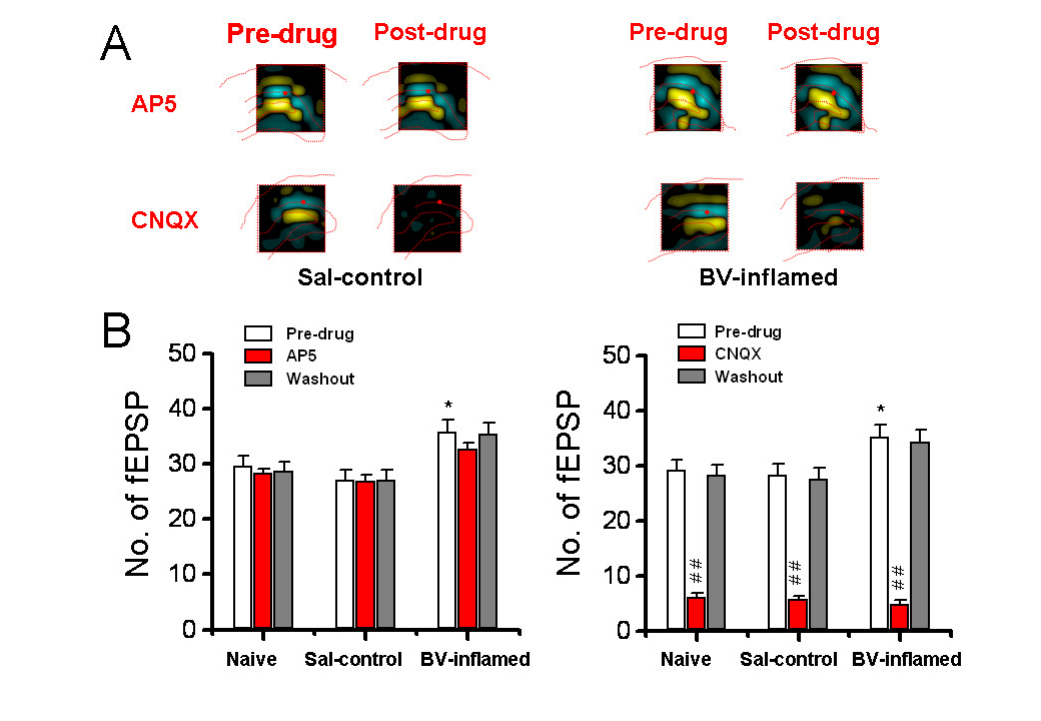
**Effects of bath application of AP5 (100 μM) or CNQX (10 μM), at 120 min after long-term potentiation induction, on the mean averaged number of field excitatory postsynaptic potential (fEPSP) that could be reliably recorded over the whole screen in naïve, saline (Sal-control), and bee venom (BV)-inflamed group of slices**. A, two-dimensional current source density imaging of changes in spatial distribution of current sources and sinks across the dentate gyrus and CA1 area before (Pre-drug) and after (Post-drug) AP5 (upper) or CNQX (lower) infusion in saline control (left) and BV-inflamed (right) groups. For other legends, see Fig. 3. B, bar histogram showing the quantification data. Note the significant increase in the number of fEPSP following BV-induced persistent pain. Application of CNQX (right) but not AP5 (left), resulted in a marked reduction of the number in each group. In the *left panel*, n = 12, 15 and 14 for naïve, saline and BV-inflamed group, respectively; in the *right panel*, n = 13, 15 and 13 for naïve, saline and BV-inflamed group, respectively. *P < 0.05 vs. Sal-control or naïve; ##*P *< 0.01, vs. Pre-drug. Error bars: ± S.E.M.

In another set of experiments, we initially probed possible mechanisms underlying BV-evoked spatial and temporal plasticity, by pharmacological blockade of NMDA and AMPA/KA receptors through bath application of AP5 (100 μM) or CNQX (10 μM) at 120 min after TBS. As for spatial plasticity, the mean number of fEPSP did not differ between pre-TBS (baseline) and post-TBS (120 min after conditioning) states, suggesting less influence of conditioning stimulation itself on the spatial property of fEPSP (data not shown). Compared with the naive and saline control group, peripheral persistent nociception resulted in a clear increase in the number of fEPSP at 120 min after LTP induction (BV-inflamed vs. saline-treated: 35.86 ± 2.04 vs. 27.20 ± 1.70, n = 14 and 15, *P *< 0.05, Fig. 12B left panel; BV-inflamed vs. saline-treated: 35.31 ± 2.12 vs. 28.33 ± 1.99, n = 13 and 15, *P *< 0.05, Fig. 12B right panel). AP5 (100 μM) failed to affect the number of fEPSP calculated in any group of slices, indicating no involvement of NMDA receptor in BV-induced enlargement of synaptic connection in the HF (Fig. 12B, left panel). In contrast, application of CNQX (10 μM) at 120 min after TBS markedly reduced the number in any group, with the inhibition rate being 77.43 ± 3.40%, 77.26 ± 3.00% and 85.23 ± 2.14% in naïve, saline- and BV-treated groups, respectively (Fig. 12B, right panel). Fig. 12A shows the 2D-CSD plots vividly presenting variable effects of the two drugs on spatial distribution of current sources and sinks in saline control (left panel) and BV-inflamed (right panel) groups. It is worthy of noting that bath infusion of CNQX (10 μM), but not AP5 (100 μM), robustly decreased the intensity of current signals around the DG and CA1 regions.

When it comes to the temporal plasticity, the maintenance of LTP was lightly but significantly blocked by AP5 (100 μM), with the inhibition rate being 7.42 ± 1.83%, 6.87 ± 2.38% and 17.21 ± 2.80% for the DG fEPSP of naïve, saline- and BV-treated group, respectively. A similar pattern of suppression was found in the case of CA1 fEPSP, with the suppression rate being 8.51 ± 1.41%, 3.48 ± 1.01% and 18.84 ± 2.03%, respectively. Bath application of CNQX (10 μM) dramatically blocked the maintenance of LTP evoked in three groups of slices. Curiously, there was no marked difference in the inhibition rate of CNQX among each group for both types of fEPSP (data not shown).

## Discussion

### Advantages of multisite electrophysiological recording using pMEA

Electrophysiological recordings with traditional microelectrodes have provided fruitful information concerning membrane properties and local neural circuitry at the single channel, single synapse and single neuron levels. However, some of the most serious limitations of the conventional glass micropipette techniques, such as limited recording site and time, typically preclude any chance of testing hypothesis regarding neural interactions as spatially extended circuits over a longer period. Substrate-integrated pMEA offers an alternative to classical electrophysiology for recording the electrical activity of cells and tissues of neuronal or cardiac origin [66]. The advantages of pMEA lies in that: (1) enable gathering large amounts of spatial information on the internal dynamics of networks with multisite recordings [65,78]; (2) long-term analysis of the spatiotemporal distribution of network level electrical activity [63,64,66]; (3) stimulating and recording the electrophysiological activity of many sites within a slice [63,65]; (4) steady recording that are less sensitive to factors such as mechanical vibrations [65,78]. Overall, the pMEA technology represents a valuable tool for stably recording electrophysiological data from multiple sites over extended periods of time from a variety of biological preparations, facilitating our understanding of complex brain operations and functions in normal and pathological states [64,68-70,79,80].

### Distinct forms of fEPSP exist between DG and CA1 area

In the present study, electrical stimulation of the PP fibers typically evoked two forms of fEPSP in rat hippocampal slices. The positive-going fEPSP was mainly distributed within the DG area, while the negative-going waveforms were largely localized in the area across stratum lacunosum-moleculare of CA1 region where apical dendrites of pyramidal cells locate [15,16,22]. This phenomenon was further validated by 2D-CSD analysis. Generally, a current source always surfaced in the DG, whereas a sink signal consistently formed in the CA1 for most of the sampling period (Fig. 3). It is well known that the PP may include two sources of origins: one belongs to polysynaptic intrahippocampal pathway originating from layer II of entorhinal area and projecting to the stratum moleculare of the DG, while the other belongs to direct intrahippocampal pathway originating from layer III of entorhinal area and targeting to the apical dendrites of the CA1 pyramidal cells [15,16,22,81-83]. Thus this result suggests that stimulation of the PP fibers could simultaneously activate multisite synaptic contacts in both DG and CA1 regions. The CA1 synaptic responses are not likely to be mediated by synaptic transmission through the DG-CA3-CA1 inter-relay, because the peak latencies of both positive-going and negative-going fEPSP were not significantly different. Therefore, it could be reasonably assumed that the positive-going fEPSP might be due to engagement of the synaptic connection between entorhinal projection and DG granular dendrites, while the negative-going fEPSP might result from direct activation of the entorhinal-CA1 pathway.

Although it has been believed that both kinds of PP fibers are glutamatergic, the glutamate receptor composition in the DG and CA1 is likely to be different according to our present pharmacological results. As shown in Fig. 5, both the DG positive-going and the CA1 negative-going fEPSP were completely blocked by CNQX, however, the latter were less sensitive to AP5 than the former, suggesting a possible difference in localization density of glutamate receptor subtypes between these two regions. This finding will be of particular interest and help to further identification of the properties of two kinds of PP fibers and their distinct targets in different subregions of the HF.

### Spatial plasticity of synaptic connection and transmission in the HF caused by persistent nociception

Mechanisms underlying spatial plasticity of synaptic connection and transmission may operate in two ways. One is simply a recruitment of presynaptic input onto a single cell, leading to gain of the fEPSP at the previously activated synaptic contacts [84]. The other possibility is an increase in the number of synaptic contacts onto more newly activated cells or an increase in postsynaptic dendritic spines on the previously activated cells, leading to enlargement of the effective network size. In the current study, we successfully recorded both types of spatial plasticity. As illustrated in Fig. 8 and 9, BV- or formalin-induced persistent nociception moved the I-O functional curves leftward, although not in a parallel manner, to those of the naïve and saline control rats. This represents an increase in synaptic efficacy due to recruitment of more inputs or activation of post-synaptic molecular and cellular events [84]. In addition, we found a new type of spatial plasticity with a distinct enlargement of neural network size probably due to increase in the number of synaptic contacts onto more newly activated cells or increase in post-synaptic dendritic spines on the previously activated cells caused by peripheral persistent nociception (Fig. 6 and 7). Taken together, these findings strongly reinforce the notion that peripheral persistent pain stimulation can really result in spatial plasticity of synaptic activity in the HF, revealed as enhanced responsiveness to electric stimuli as well as enlargement of the scope where effective fEPSP could be elicited. These spatial characteristics of synaptic plasticity, revealed by our MED64 recording system, are far beyond the scope of other classical electrophysiological recording techniques (such as the in vivo electrophysiology and in vitro patch clamp recording) and further highlight the superiority of multisite recording.

The present report also provided the first attempt to explore potential pharmacological mechanisms leading to this sort of spatial plasticity. It is of particular importance to point out that BV-evoked spatial summation of multisite synaptic responses is not due to tetanic stimulation applied to the PP fibers, nor is there any involvement of NMDA receptors in the process. Our pharmacological study showed a complete reversal of the enlarged synaptic connection by CNQX (Fig. 12), implicating a crucial role of AMPA/KA receptors in mediating nociception-induced spatial plasticity. Since the mechanisms underlying learning and memory in the HF are highly dependent on activation of NMDA receptors [84], the mechanisms for nociception-induced spatial plasticity of synaptic connection in the HF are likely to be different. However, precise mechanisms for persistent nociception-induced enlargement of synaptic connection network still remain unclear and require to be further studied.

With respect to the input pathways by which peripheral nociceptive information can be conveyed to the HF, at least two major sources are considered. One is septal-hippocampal cholinergic projection pathway which originates from medial septal nucleus and nucleus of the vertical limb of the diagonal band of Broca and passes through fornix back to both the DG granular cells and the CA1 pyramidal cells [22]. The other one is entorhinal-PP pathway *per se *which mainly receives projections from posterior parietal association cortex and anterior cingulate cortex to the entorhinal area [22]. It is known that the two cortical regions receive nociceptive information from primary somatosensory cortex which is the target of spinothalamic tract [1-3,20]. Recently, we found that BV-induced persistent pain produced long-lasting activation of mitogen-activated protein kinase subfamily members in both S1 area and HF [37,85]. Up-regulation of c-Fos protein was also predominantly localized within layer II-III of the S1 region in response to intraplantar BV injection [86]. Taken together, the spatial plasticity of synaptic connection and organization might be caused by persistent nociceptive drive produced by long-lasting activation and sensitization of primary nociceptors and spinal dorsal horn nociceptive neurons [72,73]. This hypothesis is strongly supported by the elimination of enlarged synaptic connection size (increase in number of fEPSP) in the HF by pre-blockade of nociceptive impulses in the present study (Fig. 6). In addition, the spatial plasticity may also be caused by disinhibition of inhibitory γ-aminobutyric acid (GABA) or other endogenous neuropeptides such as enkephalin, substance P, vasoactive intestinal polypeptide, cholecystokinin and somatostatin in the HF [22]. Although some reports indicate the involvement of endogenous opioid and GABA_A _receptors in nociceptive processing within the HF [87], the mechanisms underlying this hypothesis are still poorly studied and need to be further investigated using this model.

### Temporal synaptic plasticity in the HF caused by persistent nociception

Mammalian hippocampal LTP is a widely studied model of activity-dependent changes in synaptic efficacy that is assumed to provide the physiological basis for learning and memory [48-50]. A long-lasting, NMDA receptor-dependent LTP has been repeatedly elicited in the excitatory synapses made by PP fibers onto granular cells of the DG [88,89]. A few of studies have also examined the pharmacology and plasticity of the entorhinal-CA1 pathway [90,91]. In spite of these results, very limited information is available concerning LTP induction and persistence in these two pathways at the network level. In the present study, using a unique multi-electrode array recording technique, LTP of sufficient magnitude and duration could be reliably and simultaneously recorded in both pathways following TBS conditioning of the PP fibres (Fig. 1 and 2). Future experiments will be directed towards dissecting the molecular and cellular changes underlying these forms of network LTP.

A notable finding of this study was that BV- or formalin-induced inflammatory painful stimulation increased the LTP induction probability and magnitude in both DG and CA1. The enhanced LTP could be completely abolished by blocking the nociceptive inputs from the peripheral injury site (Fig. 10), suggesting a strong correlation with existence of pain. These observations are compatible with the conclusion that BV- or formalin-induced persistent pain could also produce temporal plasticity of synaptic activity in the HF, reflected as increased probability of LTP induction and augmented LTP magnitude.

The most striking feature of nociception-produced synaptic plasticity in the HF was a change in the appearance or shape of fEPSP. In almost half of the recorded slices from the BV- and formalin-inflamed group, the structure of the fEPSP waveform was remarkably deformed or tortured by persistent nociception in that it became bi- or multi-phasic at about 30-60 min after TBS conditioning, whereas the waveform of fEPSP was normally shown to be mainly single phase (Fig. 2 and 11). This was further demonstrated by the 2D-CSD plots, showing a conversion from single current source-sink dipole to binomial events after TBS in the BV-inflamed group (Fig. 3). The mechanisms underlying the deformation of fEPSPs by persistent nociception are not clear, however, the roles of septal-hippocampal cholinergic regulation of the HF should be considered, because the LTP conditioning parameter used here was TBS that mimicked the septal-hippocampal cholinergic stimulation.

### Differences in the spatial and temporal plasticity produced by formalin and BV-evoked inflammatory pain

One important point revealed by these data is the possible difference in the degree or extent of spatial and temporal plasticity elicited by BV- and formalin-induced persistent pain. As can be evidently seen from the present results, there are indeed some differences existing between these two models. For the spatial plasticity, although both models of inflammatory pain could result in a significant leftward shift of the I-O functional curves of the fEPSP amplitude or slope, the trend was rather less marked in the case of formalin-evoked persistent pain (Fig. 8 and 9). Intriguingly, no appreciable difference was found in the number of fEPSP between formalin- and BV-treated group (Fig. 6 and 7). For the temporal plasticity, formalin-induced LTP enhancement was still much smaller than that by BV injection (Fig. 10 and 11). This partial discrepancy of spatiotemporal plasticity produced by the two models of pain are in good accordance with previously reported inter-model differences in both behavioral and electrophysiological assays [72-76,92-94].

In previous studies conducted in the formalin test, it was found that most of the CA1 pyramidal cell activities were suppressed by formalin injection, however, the theta rhythmic activities were increased in the typical manner of formalin-elicited biphasic behavioral and neuronal responses [26-31]. Moreover, the expression of neurokinin 1 receptors and brain-derived neurotrophin factors in the hippocampus was also suppressed by subcutaneous injection of formalin [35,36]. The above two groups' results are in contrast to what we observed in the present study. The discrepancies are presumably ascribed to the differences in subtle experimental variables (e.g. the concentration, volume and site of formalin injection, measuring methods, observation targets and so on) but may also reflect the complexity of the higher brain structure in dealing with pain. That is, even the same kind of painful stimulus could lead to a myriad of complicated even contrasting changes in hippocampal morphology, molecular biology and physiology. Nonetheless, novel information about modulation of sensitivity, plasticity, and function of the hippocampus by painful stimuli and the gaining knowledge of the underlying mechanisms may shed new light on the roles of this limbic region in pain processing and lead to discovery of new therapeutic targets and strategies for treating not only pain but also co-morbidities of pain in the clinical setting.

In summary, a recently-developed multi-electrode array technique was successfully applied to acutely dissociated hippocampal slices in this study to demonstrate that peripheral persistent nociception could produce both spatial and temporal plasticity of synaptic connection and function in the HF. The spatial plasticity of synaptic activities is more complex than the temporal plasticity, comprising of enlargement of synaptic connection size at network level, deformed fEPSPs at local circuit level and, increased synaptic efficacy at cellular level. Finally, the multi-synaptic model established in the present study might be useful for further elucidating brain processing of emotional and cognitive aspects of pain, as well as screening novel analgesics for treating brain disorders associated with chronic pain.

## Methods

### Animals

Experiments were carried out on male albino Sprague-Dawley rats provided by Laboratory Animal Facilities of both Capital Medical University (CCMU) and the Fourth Military Medical University (FMMU). All animals were with ages of 4 weeks old (weighing 120-160 g) and were housed in groups of five per cage under controlled laboratory conditions (12 h light/12 h dark, temperature 22-26°C, air humidity 55-60%). They had free access to commercial rat pellets and tap water. The experimental procedures were approved by the Institutional Animal Care and Use Committee at both CCMU and FMMU. All animals were maintained and cared for in compliance with the guidelines set forth by the International Association for the Study of Pain [95]. The number of animals used and their suffering were greatly minimized. The experiments were blinded; all experimental rats were randomly divided into five groups: (1) naïve rats without treatment; (2) rats with subcutaneous injection of 0.9% sterile, isotonic saline solution; (3) rats with subcutaneous injection of whole BV solution; (4) rats with subcutaneous injection of formalin; and (5) rats with subcutaneous injection of 0.6 ml bupivacaine (0.25%) into the hind paw 10 min prior to ipsilateral BV treatment.

### Induction of persistent pain

Persistent pain was induced with the BV test as described previously [72]. The BV used in this study was lyophilized whole venom from *Apis mellifera *(Sigma, St. Louis, MO) dissolved in 0.9% sterile saline. A volume of 50 μl saline containing 0.2 mg BV was used during the whole experiment, because previous studies have shown that 4 μg/μl was the optimal dose to produce a prolonged pain-related behavioral response [71]. With respect to the formalin text, for each injection, 0.05 ml of 5% formalin (37.5-40% formaldehyde solution diluted in 0.9% sterile saline) was used in the present study [74,92,94]. The whole BV or diluted formalin solution was administered by subcutaneous injection into the posterior plantar surface of the left hind paw of rats [72]. The animals were carefully handled during the process to reduce the possible interruption of results caused by handling-induced stress. Intraplantar injection of the same volume of physiological saline served as the control group.

### Preparation of multi-electrode array

Procedures for the preparation of the Multi-Electrode Dish (Panasonic, MED probe) were almost the same as described by [70]. The device had an array of 64 planar microelectrodes, each 50 × 50 μm in size, arranged in an 8 × 8 pattern (inter-electrode distance, 300 μm). The microelectrode's large size resulted in lower impedance, enabling both reliable stimulation and a higher signal to noise ratio when recording. Before use, the surface of the MED64 probe was treated with 0.1% polyethyleneimine (Sigma, St. Louis, MO; P-3143) in 25 mM borate buffer (pH 8.4) overnight at room temperature. This coating helped establish sufficient adhesion of the slice to the probe surface, resulting in enough perfusion by the recording buffer (2-3 ml/min) to keep the slice healthy for more than 6 h of fEPSP recording [70]. The probe surface was rinsed three to five times with sterile distilled water before immediate use. In general, the MED64 probes could be re-used for approximately 30-40 recording sessions with a mean duration of 4-6 h. Electrode properties could be maintained constant by carefully cleaning the probe with deionized water following each recording session.

### Preparation of acute hippocampal slices

The general procedures for preparing acute hippocampal slices were similar to those described previously [68-70]. Male Sprague-Dawley rats aged 25-30 days were sacrificed by decapitation after anesthesia with 4% sodium pentobarbital (0.1 ml/100 g, i.p.) 2 h after subcutaneous saline or BV or formalin injection. Subsequently, the whole brain was rapidly removed and immediately soaked in ice-cold, oxygenated preparation buffer of artificial cerebrospinal fluid (ACSF) for approximately 1-2 min. The ACSF contained 124 mM NaCl, 3.3 mM KCl, 1.2 mM KH_2_PO_4_, 2.4 mM MgSO_4_, 10 mM glucose, 26 mM NaHCO_3_, 2.5 mM CaCl_2_, and had a pH of 7.4 adjusted by gassing with 5% CO_2_/95% O_2. _Appropriate portions of the brain were then trimmed and the remaining brain block was placed on the ice-cold stage of a vibrating tissue slicer (Dosaka, DTK-1000). Here, it deserves mentioning that all the present experiments were performed on the right (i.e. contralateral to the BV injection side) anterior hippocampus of rats in three groups, ranging from Bregma -2.52 mm to Bregma -4.08 mm according to the Atlas of the Rat Brain [96]. The stage was immediately filled with oxygenated and frozen ACSF. The thickness of each tissue slice was set at 350-400 μm. Each slice was gently taken off the blade by a writing brush, trimmed, and immediately soaked in an incubation chamber containing the oxygenated ACSF for 2 h at room temperature.

### Electrophysiological recordings

After incubation, one slice was selected and positioned on the MED64 probe in such a way that the whole HF was entirely covered by the 8 × 8 array. Once the slice settled, a netting ballast (U-shaped platinum wire with regularly spaced hair pieces) was carefully disposed on the slice to immobilize it. For the electrophysiological recordings, the probes with immobilized slices were connected to the stimulation/recording component of MED64. The slice was continuously perfused with oxygenated, fresh ACSF at the rate of 2-3 ml/min with the aid of a peristaltic pump (PERI-STAR™, WPI, USA). After a 20 min recovery of the slice, one of the 64 available planar microelectrodes was selected from the 64-switch box for stimulation following visual observation through a charge-coupled device camera connected to an inverted microscope. When not specified, monopolar, biphasic constant current pulses (30-199 μA, 0.1 ms duration) generated by the data acquisition software were applied to the PP at 0.1 Hz. Field potentials evoked at the remaining sites were amplified by the 64-channel main amplifier and then digitized at a 20 kHz sampling rate. The digitized data were displayed on the monitor screen and stored on the hard disk of a microcomputer. Five successive responses were averaged automatically in real time by the recording system. The viability of the slices was kept constant across different sets of recording sessions by measuring the threshold for evoking fEPSP of adequate amplitude.

### Experimental procedures

After selecting the best stimulation site and stabilizing the synaptic responses for about 30 min, an I-O curve was first determined for each group using the measurements of fEPSP amplitude or slope in response to a series of stimulation intensities from 30 μA to 199 μA (30 μA, 60 μA, 90 μA, 120 μA, 150 μA, 180 μA, 199 μA). Because of the technical limits of the stimulus generator, higher intensities (>199 μA) could not be applied and were not tested in the present study. The intensity of the test stimulus was then adjusted to elicit 40-60% of the maximum based on the I/O curves. Next, the stability of the whole recording system was checked by recording baseline responses for another 30 min (3 × 10 min). For LTP induction, the TBS protocol was used, which consisted of 10 bursts, each containing 4 pulses at 100 Hz with an inter-burst interval of 200 ms. It is widely accepted that such a protocol resembles in vivo conditions and has been suggested as a method to establish a link between artificial and natural synaptic activity [97]. In addition, LTP induced by such stimulation appears to be more robust and stable than that induced by other means [98]. To standardize tetanization strength in different experiments, the TBS strength was set at an intensity evoking almost half of the maximal magnitude of fEPSP. After TBS, the test stimulus was repeatedly delivered (at the identical intensity as baseline) once every 10 min for more than 2 h to allow for the observation of any changes in LTP magnitude and duration.

In experiments regarding pharmacological characterization of fEPSP, the stability was first determined by recording the baseline responses for about 30 min as described above. Then, TTX (0.5 μM and 1 μM), AP5 (50 μM and 100 μM) and CNQX (10 μM) were bath applied to separate slices at a rate of 2 ml/min. For the high magnesium-low calcium solution, the concentration of CaCl_2 _was lowered to 0.25 mM and the concentration of MgSO_4 _was raised to 4.0 mM in the ACSF. In another set of experiments, either AP5 (100 μM) or CNQX (10 μM) was bath applied at 2 h after LTP induction (a sustained peak level reached at this time) to observe their actions on spatial and temporal plasticity in the HF. In any of the above cases, complete solution exchange was achieved within 10 min of drug infusion or ionic substitution. Subsequently, fresh ACSF washed in until the drug effects vanished and the normal synaptic responses essentially recovered.

### Current source density analysis

In this study, the 2D-CSD was computed in an attempt to identify current sources and sinks in any direction within the plane of each hippocampal slice. In general, the 2-dimensional current density *I*_*m *_in the presence of a field potential Φ was given as



Since the measured field potential (Φ_*i*, *j*_) was recorded on a planar array of 64 electrodes, the second partial derivatives at the center of particular electrodes could be computed from the measured field potential on that electrode and its neighbors as



By considering the medium as ohmic with homogeneous conductance (*σ*_*X *_= *σ*_*Y *_= *σ*), and under the assumption of equidistant electrodes (Δ_*X *_= Δ_*Y *_= Δ), the normalized CSD (I*_i, *j*_) could be defined and computed as



With the normalized CSD values at the center of the electrodes, it became possible to compute the density at any point (x, y) within the 8 × 8 array using bilinear interpolation. After all of the above calculations, we used the color yellow to represent positive currents (sources), the color blue to represent negative currents (sinks), and the color black to map zero current. Finally, CSD images at selected time points were plotted across all 64 recording sites for each group of slices.

### Drugs

All drugs were purchased from Sigma-Aldrich and, except for CNQX, were dissolved in deionized water as stock solutions for frozen aliquots. They were diluted to the desired concentration in ACSF before immediate use. CNQX was dissolved in dimethylsulfoxide (DMSO, final concentration 0.1%) and prepared as described above.

### Offline analysis

For quantification of the I-O relationship, the amplitude and slope of fEPSP were analyzed off line by the MED64 Conductor. For LTP data, the amplitude and slope of evoked fEPSP were normalized and expressed as a percentage of the averaged value measured during the last 10 min baseline period. Evaluations of drug effects were carried out on the basis of the difference between the pre-drug recording and the 10 min after drug infusion (when the drug effect was most potent). The total number of effective fEPSP (> 20% baseline) reliably recorded over the HF was counted by an experimenter unaware of the experimental design and averaged across slices for each group. Data sets included results from only one slice per rat (n = number of slices). All data were presented as mean ± S.E.M. When necessary, the statistical significance was determined using either the Student's *t *test (paired and two-independent sample) or one-way ANOVA (post-hoc Fisher's PLSD). The level of *P *< 0.05 was assumed as statistically significant.

## Competing interests

The authors declare that they have no competing interests.

## Authors' contributions

X-YZ, M-GL, D-LY and Y-W mainly, YH and D-DW partially collected the primary data; X-FC established the 2D-CSD analysis method and conducted the imaging transformation work; F-KZ and HL helped in MED64 system setup and slice preparation; JC and X-SH designed the experiments; JC and M-GL wrote the manuscript. All authors have read and approved the manuscript finally.
